# Flood-derived sludge as a reservoir of viable opportunistic pathogens after the October 2024 DANA in Valencia (Spain)

**DOI:** 10.1007/s10123-026-00821-4

**Published:** 2026-04-16

**Authors:** Marina Pérez-Lara, Pablo Ibáñez-Payá, Manuel Ruiz-Platón, Héctor Carmona-Salido, José Juan Mateo, Carmen Amaro, Rubén Salvador-Clavell

**Affiliations:** 1 Instituto Universitario de Investigación en Biotecnología y Biomedicina, Universitat de València, Burjassot, Spain; 2 Departamento de Microbiología y Ecología, Universitat de València, Burjassot, Spain

**Keywords:** DANA, Flood-derived sludge, Enterobacterales, Opportunistic pathogens, Public health risk

## Abstract

Extreme flooding events generate large volumes of contaminated sludge that may act as environmental reservoirs of viable microorganisms, yet their capacity to harbour viable bacteria with pathogenic potential remains poorly understood. Following the October 2024 DANA event in Valencia (Spain), flood-derived sludge was collected one month after the episode and analysed using enrichment-based culture, molecular detection, and phenotypic characterization. High loads of culturable bacteria (> 10⁶ CFU·g⁻¹ after enrichment) were recovered, with microbial communities dominated by Enterobacterales, together with *Aeromonas* spp. and *Pseudomonas* spp., consistent with contamination from sewage overflows and disturbed urban environments. Culture-based approaches did not yield *Vibrio* spp.; however, weak qPCR signals for *Vibrio vulnificus* suggest its presence at low abundance and/or in a viable but non-culturable state. Species-level identification revealed the presence of opportunistic pathogenic taxa, including *Escherichia coli*, *Enterobacter cloacae*, *Salmonella* spp. and *Aeromonas* spp. Functional characterization showed widespread proteolytic activity and biofilm formation among isolates. In selected strains, haemolytic activity, resistance to human serum, and multidrug resistance were also observed. Notably, one E. coli isolate combined multiple virulence-associated traits and antibiotic resistance, highlighting its potential clinical relevance. Overall, these results demonstrate that flood-derived sludge deposited after extreme rainfall events can function as a reservoir of viable bacteria with pathogenic potential. This study highlights the importance of incorporating microbiological surveillance and risk-oriented approaches into post-flood management strategies under a One Health framework.

**Supplementary Information:**

The online version contains supplementary material available at 10.1007/s10123-026-00821-4.

## Introduction

Climate change is increasing the frequency and intensity of extreme hydrometeorological events in the Mediterranean, such as the “cold drop” (DANA), which causes catastrophic flash floods (AEMET 2025; Erickson et al. 2019). In late October 2024, an exceptional DANA affected Valencia, Spain, with rainfall exceeding 770 L·m⁻², resulting in 229 fatalities and severe environmental damage (ORAIN 2025). Significant impacts were also observed in the Albufera Natural Park, located within the affected area, where massive sludge and debris accumulation threatened the wetland’s ecological balance. Beyond physical destruction, these floods profoundly alter microbial communities. Intense runoff mobilizes organic matter and industrial contaminants, while the collapse of sanitation infrastructure facilitates the release of untreated wastewater (Cann et al. 2013). This substantially increases environmental microbial loads and public health risks, posing a long-term threat to both ecosystem integrity and human safety (Ahern et al. 2005; Sinigalliano et al. 2007).

In this context, flood-derived sludge and sediments constitute highly concentrated matrices in which microorganisms may persist for extended periods, acting as environmental reservoirs capable of sustaining secondary contamination and long-term sanitary risks (Abraham and Wenderoth 2005; Sinigalliano et al. 2007; Pow et al. 2025). Several studies have demonstrated that post-flood sediments can harbour viable bacteria even months after the initial event, thereby increasing human and animal exposure to opportunistic pathogens (Kelman et al. 2011; Bich et al. 2011).

Among the microorganisms of greatest public health relevance in these scenarios are members of Enterobacterales. This family includes clinically relevant genera such as *Escherichia*,* Salmonella*,* Shigella*,* Klebsiella* and *Enterobacter*, which are responsible for millions of gastrointestinal and systemic infections worldwide each year (Lynch and Shaman 2023). The abundance of faecal indicator bacteria, such as *E. coli* and intestinal enterococci in environmental matrices is widely accepted as a robust indicator of faecal contamination and sanitation system failure, and their abundance has been shown to increase markedly following heavy rainfall and flooding events (Kelman et al. 2011). In addition, previous studies have reported the presence of *Vibrio* spp. in sludge and flood-affected environments, highlighting their potential relevance in these ecological niches (Mas-Coma et al. 2025).

Despite growing awareness of flood-associated microbial hazards, the role of deposited sludge as a persistent reservoir of viable opportunistic bacteria remains insufficiently characterized. Many post-disaster studies rely primarily on molecular detection of bacterial DNA, which does not necessarily reflect microbial viability or pathogenic potential (Sinigalliano et al. 2007). Consequently, there is a need for integrated approaches combining culture-based methods, molecular identification, and functional characterization to more accurately assess the real epidemiological risk associated with post-flood sediments (Cann et al. 2013).

In response to public concern following the October 2024 DANA, sedimented flood sludge samples were collected from the dry riverbed called Rambla del Poyo in Paiporta, one of the areas most severely affected by the flooding. The objective of this study was to evaluate whether flood-derived sludge acts as a reservoir of viable Enterobacterales and other opportunistic bacteria one month after the event, and to characterize the recovered isolates in terms of virulence-associated traits, biofilm-forming capacity, human serum resistance, and antimicrobial susceptibility. The results provide relevant data for post-disaster environmental surveillance and contribute to improved risk assessment strategies in the context of increasingly frequent extreme climatic events.

## Materials and methods

### Sampling and sample handling

**Fig. 1:**
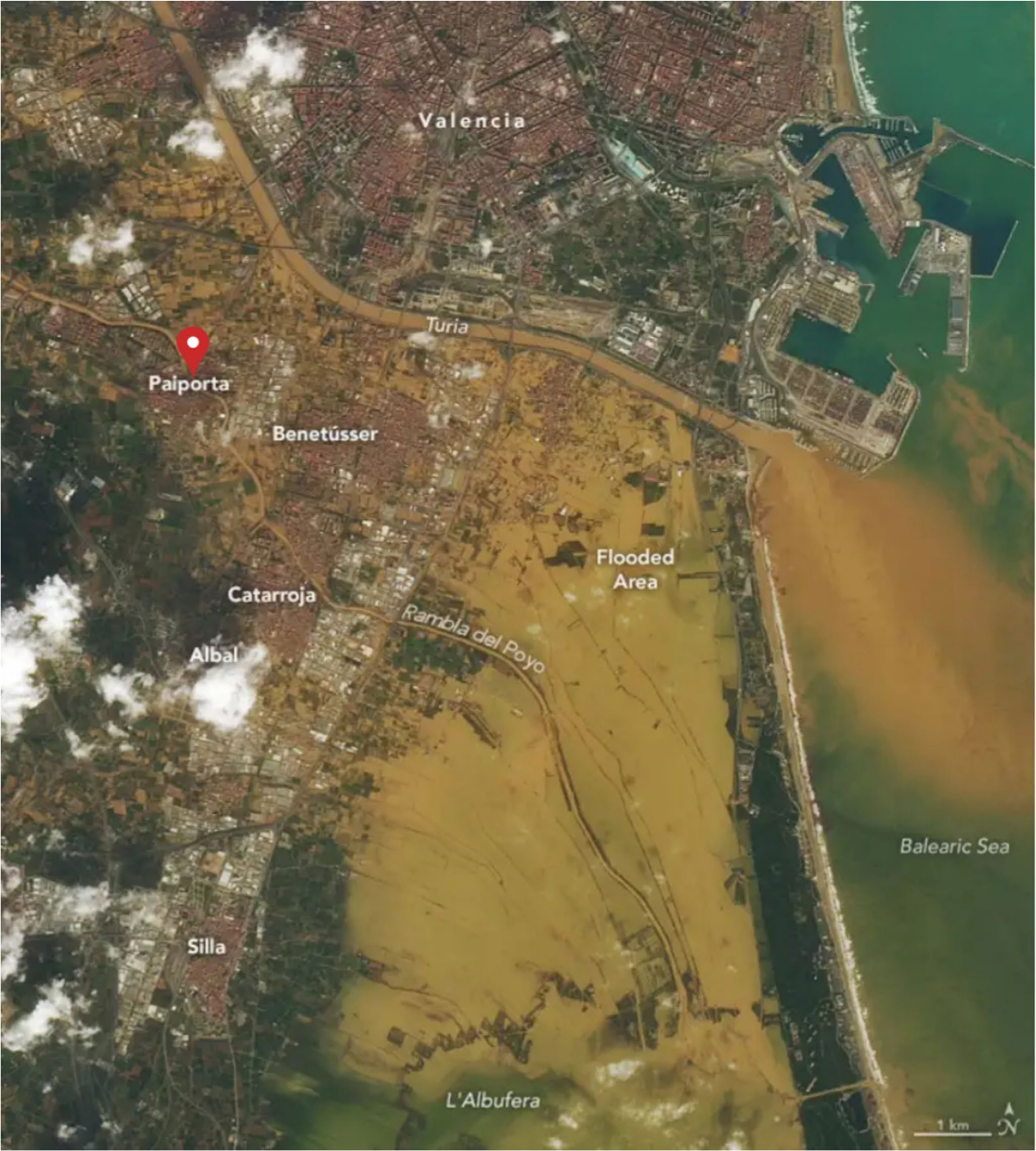
Sampling location in Rambla del Poyo in Paiporta. The marked point corresponds to the coordinates: 39°25’37.8"N 0°24’49.2"W. Image acquired the day after the flood, 30th October, adapted from NASA Earth Observatory by Lauren Dauphin, using Landsat data from the U.S. Geological Survey

Three sludge samples were aseptically collected from the Rambla del Poyo in Paiporta (Valencia, Spain), approximately one month after the flooding event (December 4th) (Fig. 1). Temperature was recorded in situ and samples were transported to the laboratory under refrigerated conditions, stored at 4 °C, and processed within 24 h.

### Microbial isolation and quantification after enrichment

Each sludge sample was processed in triplicate to examine faecal contamination and the presence of *Vibrio* spp. The inclusion of *Vibrio* spp. was based on the geographical impact of the DANA event, which affected areas adjacent to the Albufera coastal lagoon, a known habitat for species such as *V. vulnificus* (Salvador-Clavell et al. 2025). Floodwaters may facilitate the transport of these bacteria from saline or brackish environments into inland sediments, justifying their inclusion in the microbial risk assessment (Esteves et al. 2015). To this end, enrichment steps targeting either enterobacteria or vibrios were performed using the Enterobacteriaceae Enrichment (Mossel formulation) and Alkaline Peptone Water with 1.5% NaCl broths, respectively (Shepelin et al. 2017; Quintero-Campos et al. 2025).

To this end, one gram of sludge was resuspended in 100 mL of enrichment broths and incubated at 37 °C for enterobacteria or 28 °C for vibrios. Culturable bacteria (calculated and expressed as colony-forming units per gram of sludge (CFU·g⁻¹, wet weight)) were counted at 0 and 24 h of incubation by plating appropriate dilutions prepared in phosphate-buffered saline (PBS; pH 7.0) on the general medium TSA (Tryptic Soy Agar) for enterobacteria and TSA-1 (supplemented with NaCl 0.5% [w/v]) for *Vibrio* spp., both used as non-selective media, and on selective media including MCA (MacConkey agar) and HEA (Hektoen Enteric agar) for enterobacteria, and TCBS (Thiosulfate–Citrate–Bile Salts–Sucrose agar) for *Vibrio* spp. (all media purchased from Condalab, Spain).

For isolation purposes, colonies displaying presumptive morphologies on selective media were selected and subsequently purified on TSA (enterobacteria) or TSA-1 for presumptive *Vibrio* isolates, yielding a total of 30 isolates for further characterization. Each pure isolate was stored at −80 °C in LB or LB-1 (Luria-Bertani broth; Condalab) supplemented with 20% (v/v) glycerol until further used.

### Viable DNA detection and quantification from enrichment broth

Genomic DNA was extracted from enrichment broths 24 h post-inoculation using the DNeasy PowerSoil Pro Kit (Qiagen, Germany), following the manufacturer’s instructions. Quantitative PCR (qPCR) assays were subsequently performed to detect and quantify selected members of the order Enterobacterales, including *Escherichia coli*, *Salmonella* spp., *Enterobacter cloacae*, *Klebsiella* spp., *Shigella* spp., and *Proteus vulgaris*, as well as pathogenic *Vibrio* species such as *Vibrio vulnificus*, *V. cholerae*, and *V. parahaemolyticus*. These taxa were selected based on their previous detection in nearby environments (Carmona-Salido et al. 2025; Esteves et al. 2015; Salvador-Clavell et al. 2025).

For the identification and characterization of *V. cholerae*, three molecular markers were used to distinguish species identity, serogroup O1, and toxigenic potential. Specifically, the species-specific gene *viuB* was used for confirmation of *V. cholerae*, the *rfbO1* gene for identification of serogroup O1 strains, and the *ctxA* gene for detection of cholera toxin-producing strains (Garrido-Maestu et al. 2014; Nasreen et al. 2020). This strategy allowed the differentiation of *V. cholerae* at both the taxonomic and functional levels.

qPCR reactions were performed using 25 ng of template DNA and SYBR Green Master Mix (Applied Biosystems, UK), with each primer at a final concentration of 1 µM. Primer sequences and expected amplicon sizes are listed in Supplementary Table 1. Amplifications were carried out in a final volume of 20 µL using a StepOnePlus™ Real-Time PCR System (Applied Biosystems). The thermal cycling conditions consisted of an initial denaturation at 95 °C for 10 min, followed by 40 cycles of 95 °C for 15 s and 60 °C for 1 min. A melting curve analysis was performed at the end of each run.

All reactions were performed in technical triplicate. Reference strains used as positive controls were obtained from the Spanish Type Culture Collection (CECT) and included: *Salmonella* sp. CECT 443, *E. coli* CECT 101, *Enterobacter cloacae* CECT 194, *Klebsiella pneumoniae* CECT 143, *Proteus vulgaris* CECT 165, *Shigella sonnei* CECT 4887^T^, *V. cholerae* O1 CECT 652, *V. cholerae* non-O1 CECT 8265, *V. vulnificus* CECT 4999, and *V. parahaemolyticus* CECT 511^T^.

### Identification of bacterial isolates

Identification of bacterial isolates was performed using a stepwise approach combining molecular and phenotypic methods. Partial 16 S rRNA gene sequencing was initially carried out, and taxonomic assignment was based on sequence similarity thresholds (≥ 99% for species-level and ≥ 97% for genus-level identification). When sequencing results were inconclusive, species-specific PCR assays were applied. For isolates that remained unresolved, biochemical identification was performed using API systems (bioMérieux), including API 20E and API 32E for Enterobacterales and API 20NE for non-enteric Gram-negative bacteria. Identification confidence was interpreted according to the manufacturer’s criteria. In cases where API profiles suggested either Aeromonas or Vibrio, additional differentiation was performed using O/129 susceptibility testing.

For genetic identification, the partial 16S rRNA gene was amplified (616V: 5’-AGAGTTTGATYMTGGCTCAG-3’ and 699R: 5’-RGGGTTGCGCTCGTT-3’ primers) and sequenced by Sanger (Arahal et al., 2008). Sequencing was carried out at the *Servei Central de Suport a la Investigació Experimental* (SCSIE, University of Valencia, Valencia, Spain). Forward and reverse reads were assembled into consensus sequences using UGENE v52.1 (Okonechnikov et al. 2012). Taxonomic assignment was performed by comparison of the consensus sequences against NCBI 16 S ribosomal RNA sequence database (Bacteria and Archaea) using the BLASTN algorithm (https://blast.ncbi.nlm.nih.gov/Blast.cgi).

Although partial 16 S rRNA gene sequencing allows reliable taxonomic assignment in many cases, its resolution may be limited for closely related taxa, particularly within Enterobacterales and Vibrionaceae. In these cases, additional molecular and biochemical approaches were employed to improve taxonomic resolution and to confirm species-level identification. PCR reactions were carried out using a commercial Master Mix containing Speedy DNA polymerase (NYZtech, Portugal), with 10 ng of template DNA per reaction. Primer sequences, annealing temperatures, extension times, and expected amplicon sizes are detailed in Supplementary Table 2. Positive controls used in these assays included *Aeromonas caviae* CECT 4222, *A. hydrophila* CECT 5173, *A. media* CECT 4232^T^, *A. veronii* CECT 4257^T^, *E. coli* CECT 101, and *Enterobacter cloacae* CECT 194.

In addition, isolates identified as *E. coli* were further screened by PCR for the presence of genes associated with the main pathogenic enterotypes, including enteroadherent, enterorrhagic, enteroinvasive, enteropathogenic, and enterotoxigenic strains (Genes and primers are listed in Supplementary Table 2).

For phenotypic identification we used API strips (20E, 20NE and 32E) following the manufacturer’s instructions and the oxidase test using commercial oxidase test strips (Sigma-Aldrich, USA). In all cases, bacterial suspensions were prepared from overnight cultures and resuspended in sterile saline solution (0.85% NaCl), adjusting turbidity to a 0.5 McFarland standard. These suspensions were used to inoculate the API strips following the manufacturer’s instructions. The strips were incubated according to the manufacturer’s recommendations for 24 h, and the biochemical profiles were interpreted using the APIweb™ database. Oxidase activity was considered positive when a dark purple coloration developed within 10–30 s. Sensitivity to the vibriostatic agent O/129 was assessed using commercial disks containing 150 µg of O/129 (Oxoid). Disks were placed on the surface of TSA or TSA-1 agar plates previously inoculated with the bacterial suspension, and plates were incubated at 37 °C for 18–24 h. *V. vulnificus* CECT 4999 and *A. hydrophila* CECT 5173 were used as positive and negative controls. Isolates showing susceptibility to O/129 were considered consistent with *Vibrio* spp., including *V. fluvialis*, whereas resistance to O/129 was interpreted as indicative of *Aeromonas* spp., according to established diagnostic criteria.

### Virulence-related phenotypic assays

To functionally characterize the virulence-related traits of the isolates, we first evaluated extracellular enzymatic activities and biofilm-forming capacity as initial screening assays. These assays were selected to capture key traits associated with environmental persistence and host interaction. Based on these results, isolates displaying the most relevant virulence-associated phenotypes were selected for further characterization, including resistance to human serum complement, as an indicator of potential survival in host-associated conditions and increased invasive potential.

#### Extracellular activities

After overnight growth on TSA or TSA-1 plates at 37 °C, each isolate was inoculated into tubes containing LB3 (LB/LB-1 supplemented with 3% [w/v] gelatin; Panreac, Spain) using a straight-wire stab, and onto TSA or TSA-1 plates supplemented either with 10% (v/v) skim milk (casein plates) or 2.5% (v/v) sheep blood (Thermo Fisher Scientific, UK) (haemolysis plates). Tubes and plates were incubated at 37 °C for 24 h, after which the tubes were chilled on ice for 30 min. Liquefaction of the medium in LB3 tubes or the presence of a clear transparent halo surrounding the colonies on casein and haemolysis plates was interpreted as a positive result.

#### Biofilm production

Biofilm formation was quantified using a crystal violet staining assay in 96-well polystyrene microplates (Carmona-Salido et al. 2025). Overnight cultures grown in LB broth were diluted 1:100 in fresh medium and used to inoculate the wells. Plates were incubated statically at 37 °C for 24 h. After incubation, wells were gently washed with PBS, fixed with 100% methanol (Thermo Fisher Scientific, Spain), and air-dried. Biofilms were stained with 0.1% (w/v) crystal violet, washed to remove excess stain, and air-dried again. The bound dye was solubilized with 30% (v/v) acetic acid (Thermo Fisher Scientific), and absorbance was measured at 600 nm (OD₆₀₀).

#### Human serum resistance

Resistance to complement complex present in human blood serum was evaluated in selected isolates exhibiting high extracellular enzymatic activity or high biofilm biomass. Isolates were grown to stationary phase in LB and diluted to an initial concentration of 10³ CFU·mL⁻¹ in commercial male AB type human serum (Sigma-Aldrich, Spain). Serum inoculated with PBS was used as a sterility control. Suspensions were incubated at 37 °C for 4 h. Viable counts were determined by drop plating on TSA at 0 and 4 h. Survival was expressed as the percentage of viable cells relative to the initial inoculum (Hernández-Cabanyero et al. 2019).

### Antibiotic resistance

Isolates previously analysed for serum resistance were grown overnight (37 °C, 120 rpm) and spread onto Mueller–Hinton agar plates (Condalab) containing 0.5% NaCl. Commercial antibiotic disks (listed in Supplementary Table 3) were applied to the plates, which were then incubated at 37 °C for 24 h. Inhibition zones were measured and interpreted according to EUCAST guidelines. The antibiotic panel was selected based on its clinical relevance for Gram-negative bacteria, particularly those species identified or presumptively classified in this study.

### Statistical analysis and data representation

All experiments were performed in triplicate. Normality and homoscedasticity were assessed using the Shapiro–Wilk and Levene’s tests, respectively. Differences between groups were evaluated using one-way analysis of variance (ANOVA), followed by Tukey’s honestly significant difference (HSD) post hoc test when appropriate. Statistical analyses were performed using RStudio (Posit Team 2025).

## Results

### Determination of bacterial load in the sludge

At the sampling site, the air temperature was 18.3 °C. At the initial time point, the highest bacterial counts were recovered on TSA, reaching a maximum density of 4 × 10⁶ CFU·g⁻¹. After 24 h of enrichment, counts increased significantly to values exceeding 10¹⁰ CFU·g⁻¹ (*p* < 0.05).

**Fig. 2:**
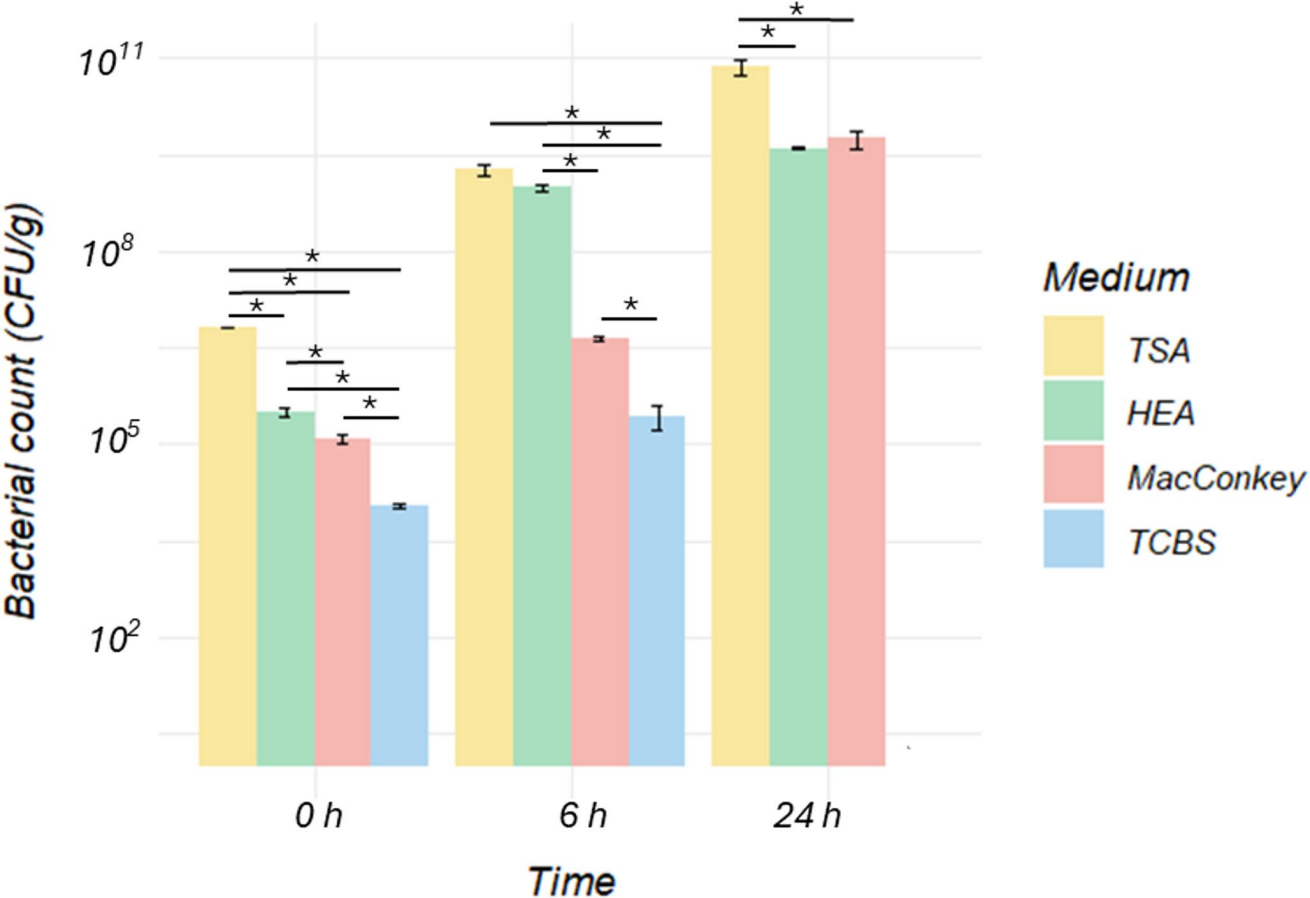
Determination of bacterial load in sludge using general and selective media. Assumptions of normality and homogeneity of variances were met (Shapiro–Wilk test, p > 0.05; Levene’s test, p > 0.05). Differences among media at each incubation time were analysed using one-way ANOVA followed by Tukey’s post hoc test, revealing statistically significant differences (p < 0.05)

Bacterial counts (CFU·g⁻¹) after 24 h of enrichment in Enterobacteriaceae broth are shown in Fig. 2. Across all enrichment media, bacterial densities increased over time, reaching their highest values at 24 h. Among the selective media for Enterobacterales, HEA yielded higher recoveries than MCA (Fig. 2).

In contrast, no growth was detected in APW-1.5, the enrichment broth for vibrios, and consequently, no colonies were observed on TCBS agar after 24 h of incubation.

### Quantitative PCR detection of potentially pathogenic bacteria in sludge samples

Potentially pathogenic Enterobacterales and *Vibrio* spp. were detected and quantified after enrichment in Enterobacteriaceae broth for 24 h by qPCR (Table 1). Among Enterobacterales, *E. coli*, *Salmonella* sp., *Shigella* sp., *Enterobacter cloacae*, *Klebsiella* sp., and *P. vulgaris* were detected.

**Table 1:** Quantitative PCR results for potentially pathogenic Enterobacterales and ***Vibrio*** spp. after 24 h-enrichment. Cycle threshold (Ct) values obtained at 24 h of enrichment are shown

Bacterial species	qPCR values^a^
*E. coli*	18.61 (+++)
*Salmonella* sp.	16.97 (+++)
*Shigella* sp.	29.85 (+)
*Enterobacter cloacae*	23.47 (++)
*Klebsiella* sp.	23.52 (++)
*V. vulnificus*	34.52 (+/-)
*V. cholerae* ^*b*^	NA
*V. parahaemolyticus*	NA

a. Results in parenthesis indicated a qualitative classification: **+++**, high abundance (Ct ≤ 20); **++**, moderate abundance (Ct > 20 and ≤ 25); **+**, low abundance (Ct > 25 and ≤ 30); **+/-**, very low abundance or weak detection (Ct > 30 and ≤ 35); **−**, not detected or Ct > 35. NA, non-amplified

b. For the identification and characterization of *Vibrio cholerae*, three molecular markers were selected to distinguish species identity (*viuB*), serogroup O1 (*rfbO1)*, and toxigenic potential (*ctxA*). No amplification was obtained for any of the three genes

In contrast, detection of *Vibrio* spp. was limited, with most assays yielding non-detectable or weak signals (Ct > 35) consistent with absence or very low abundance. The only exception was *V. vulnificus*, which was detected at very low levels (Table 1).

### Isolation and identification of viable bacteria

After several rounds of purification on TSA, a total of 30 isolates were analysed in this study. Based on partial 16 S rRNA gene sequencing, approximately 90% of the isolates were assigned to the genus level (Supplementary Table 4), including *Citrobacter*, *Enterobacter*, *Aeromonas*, and *Pseudomonas*. These genera are commonly found in aquatic environments.

To improve taxonomic resolution, additional conventional PCR assays targeting species- or genus-specific genetic markers were performed (Table 2). Using this approach, *Aeromonas hydrophila/caviae/sobria*, *Escherichia coli*, and *Enterobacter cloacae* were identified. PCR-based screening of *E. coli* isolates for major pathogenic enterotypes yielded negative results in all cases, suggesting that these strains are unlikely to cause enteric infections. Nevertheless, the identification of *E. coli* at both the genetic and phenotypic levels indicates possible faecal contamination and is therefore of particular relevance from a public health perspective.

**Table 2:** Identification of the selected isolates based on the most informative molecular and biochemical method

Isolate	Identification	Method^a^
1	*Citrobacter* spp.	16 S rRNA sequencing
2	*Pseudomonas* spp.	16 S rRNA sequencing
3	*Pseudomonas* spp.	16 S rRNA sequencing
4	*Aeromonas hydrophila/caviae/sobria*	API 20E (3046127) API 32E (30677721010) + O/129
5	*A. hydrophila*	species-specific PCR
6	*A. media*	species-specific PCR
7	*E. coli*	species-specific PCR
8	*E. coli*	species-specific PCR
9	*Citrobacter freundii*	16 S rRNA sequencing
10	*A. hydrophila/caviae/sobria*	API 20E (3046127) API 32E (30677721010) + O/129
11	*A. hydrophila/caviae/sobria*	API 20E (3046127) API 32E (30677721010) + O/129
12	*Pseudomonas* spp.	16 S rRNA sequencing
13	*Pseudomonas* spp.	16 S rRNA sequencing
14	*P. stutzeri*	API 20NE (1044675)
15	*Citrobacter freundii*	API 20E (1604573)
16	*A. caviae*	species-specific PCR
17	*Acinetobacter junii/johnsonii*	API 20NE (1040051)
18	*Enterobacter cloacae*	species-specific PCR
19	*A. junii/johnsonii*	API 20NE (1040051)
20	*E. coli*	species-specific PCR
21	*E. coli*	species-specific PCR
22	*Citrobacter freundii*	API 20E (1604573)
23	*Enterobacter* spp.	16 S rRNA sequencing
24	*Citrobacter* spp.	16 S rRNA sequencing
25	*Citrobacter freundii*	API 20E (1604573)
26	*E. coli*	species-specific PCR
27	*E. coli*	species-specific PCR
28	*Aeromonas* spp.	16 S rRNA sequencing
29	*Pseudomonas* spp.	16 S rRNA sequencing
30	*P. stutzeri*	API 20NE (1044675)

^a^ API 20E/20NE/32E identification: parenthesis indicate the corresponding biochemical profile. Isolates 4, 10 and 11 were additionally subjected to the vibriostatic agent O/129 susceptibility test to discriminate between *Aeromonas* and *Vibrio*. All isolates were resistant to O/129, supporting their classification as *Aeromonas* spp

In parallel, biochemical profiling using API 20E and API 20NE systems enabled the identification of *Pseudomonas stutzeri*, *Citrobacter freundii*, and *Acinetobacter* spp., isolates that could not be conclusively identified by 16 S rRNA sequencing or species-specific PCR. Three additional isolates yielded ambiguous biochemical profiles compatible with either *Aeromonas* spp. or *Vibrio fluvialis*; this ambiguity was resolved using the O/129 susceptibility test. All three isolates were resistant to O/129 and were therefore classified as *Aeromonas* spp.

Overall, species-level identification was achieved for approximately 60% of the isolates. Table 2 summarises the identification results and indicates, for each isolate, the method that provided the highest taxonomic resolution.

### Haemolytic and proteolytic activities

Extracellular activity assays revealed marked heterogeneity among the 30 isolates tested (Table 3). Haemolytic activity was detected in three isolates (10%), all of which exhibited complete haemolysis (β-haemolysis) at both 24 and 48 h, whereas the remaining isolates showed no haemolytic activity (γ-haemolysis). Overall, proteolytic activity was observed in 14 isolates (47%), either against casein (13 isolates, 43%), gelatine (8 isolates, 27%), or both substrates simultaneously (6 isolates, 20%). Some isolates exhibited activity against both substrates, and therefore percentages are not additive.

**Table 3:** Extracellular activities of bacterial isolates. Haemolytic and proteolytic (casein or gelatine) activities were evaluated on isolates. Positive results (+) were defined as the presence of a clear halo surrounding the colonies on casein or haemolysis plates, or liquefaction of the medium in gelatin tubes. Negative results (−) indicate absence of detectable activity under the experimental conditions

Isolate	Haemolysis		Casein hydrolysis		Gelatine hydrolysis
	24 h	48 h	24 h	48 h	24 h
1	-	-	-	-	-
2	-	-	-	-	-
3	-	-	-	-	-
4	+	+	+	+	+
5	-	-	+	+	-
6	-	-	+	+	-
7	-	-	+	+	-
8	-	-	-	-	-
9	-	-	-	-	-
10	-	-	+	+	+
11	-	-	+	+	+
12	-	-	-	-	-
13	-	-	+	+	-
14	-	-	-	-	-
15	-	-	-	-	-
16	-	-	+	+	+
17	-	-	+	+	+
18	-	-	-	-	-
19	-	-	-	-	-
20	+	+	-	-	-
21	-	-	+	+	-
22	-	-	-	-	-
23	-	-	-	-	-
24	-	-	+	+	-
25	-	-	-	-	-
26	-	-	+	+	-
27	-	-	+	+	-
28	+	+	+	+	+
29	-	-	-	-	-
30	-	-	-	-	-

### Biofilm production

**Fig. 3:**
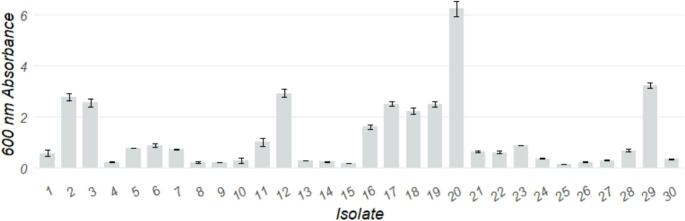
Biofilm production of bacterial isolates. Biofilm formation was measured by absorbance at 600 nm after crystal violet staining. Bars represent mean values ± standard deviation of three replicates

Biofilm formation varied widely among the isolates (Fig. 3). Most isolates displayed low to moderate biofilm production, whereas a subset of isolates (2, 3, 12, 16–19, 20, and 29) showed strong biofilm-forming capacity. Isolate 20, identified as *E. coli*, exhibited the highest biofilm biomass, reaching an OD₆₀₀ value of approximately 6.

### Human serum resistance

Based on extracellular activity and biofilm formation, three isolates (4, 20 and 28) were selected for serum resistance assays. Human serum resistance was included as a functional assay because survival in serum reflects the ability of Gram-negative bacteria to withstand complement-mediated killing, a trait that is closely associated with invasive and septicaemic potential. Among all tested isolates, only isolate 20 resisted and grew in human serum, with a proliferation rate exceeding 3,000% (3292.13 ± 460.35%) after 4 h of exposure. Isolates 4 and 28 did not survive under the tested conditions. These results suggest that isolate 20 displays phenotypic traits associated with increased invasive potential.

### Antibiotic resistance

Antibiotic susceptibility testing of the three selected isolates is shown in Table 4. Isolate 20 exhibited the lowest overall susceptibility, showing resistance to aztreonam, ceftazidime, ciprofloxacin, ertapenem, trimethoprim/sulfamethoxazole and piperacillin, as well as intermediate susceptibility to cefoxitin. Isolate 4 showed resistance to ciprofloxacin and trimethoprim/sulfamethoxazole, and intermediate susceptibility to aztreonam and ceftazidime. In contrast, isolate 28 was susceptible to all antibiotics tested.

**Table 4:** Antibiotic susceptibility profiles of selected bacterial isolates using the diffusion agar method. Intrinsic resistance patterns characteristic of certain genera were considered in the interpretation of antibiotic susceptibility results to avoid misclassification of intrinsic resistance as acquired resistance

Antibiotic^a^	Antibiogram results for^b^:		
	Isolate 20	Isolate 4	Isolate 28
Amoxicillin/Clavulanic acid (20/10 µg)	19.67 ± 0.58 (S)	NT	NT
Aztreonam (30 µg)	17.00 ± 0.00 (R)	27.00 ± 0.00 (I)	35.00 ± 0.00 (S)
Cefoxitin (30 µg)	16.00 ± 0.00 (I)	NT	NT
Ceftazidime (30 µg)	16.33 ± 0.58 (R)	21.00 ± 0.00 (I)	29.00 ± 1.73 (S)
Ciprofloxacin (5 µg)	17.33 ± 0.58 (R)	22.33 ± 1.53 (R)	32.67 ± 2.89 (S)
Ertapenem (10 µg)	18.5 ± 0.58 (R)	NT	NT
Netilmicin (30 µg)	18.33 ± 0.58 (S)	NT	NT
Piperacillin (100 µg)	20.00 ± 1.00 (R)	NT	NT
Trimethoprim/Sulfamethoxazole (25 µg)	15.00 ± 1.00 (R)	14.67 ± 0.58 (R)	19.67 ± 2.08 (S)

^a^ Quantity of antibiotic in the disk is indicated in parenthesis

^b^ Inhibition zone diameters (mm) were determined by disk diffusion on agar plates and values are expressed as mean ± standard deviation. Interpretation as Resistant (R), Susceptible (S) or Intermediate (I) was performed according to EUCAST (2025) breakpoints for *Escherichia coli* and *Aeromonas* spp. NT: non-tested

## Discussion

Extreme flooding events are increasingly recognised as drivers of environmental and public health disturbances, yet the microbiological consequences of sediment deposition remain insufficiently explored (Ahern et al. 2005; Erickson et al. 2019). In particular, flood-derived sludge represents a highly concentrated matrix in which microorganisms may persist and accumulate, acting as potential reservoirs of contamination and secondary exposure (Abraham and Wenderoth 2005; Sinigalliano et al. 2007; Pow et al. 2025). In this study, we show that sludge collected one month after a major DANA event in Valencia harbours high loads of culturable bacteria and a diverse set of opportunistic taxa with relevant virulence-associated traits.

A key finding of this study is that, following selective enrichment, flood-derived sludge samples supported the growth of a subset of culturable bacteria, predominantly belonging to Enterobacterales, together with *Aeromonas* and *Pseudomonas*. (Bekkelund et al. 2023). These taxa are well-recognized components of aquatic environments and are commonly considered part of the environmental microbiota. In this study, *Pseudomonas* isolates were not consistently identified at the species level and are therefore reported at the genus level. However, these genera are also recognized as opportunistic pathogens capable of causing infections under favourable conditions, particularly in immunocompromised hosts (Ihssane et al. (2023). Therefore, their detection in flood-derived sediments should be interpreted in terms of potential risk rather than direct evidence of pathogenicity.

This microbial composition is consistent with contamination derived from sewage overflows and urban runoff, which are well-documented consequences of extreme rainfall events (Cann et al. 2013; Sinigalliano et al. 2007). Although enrichment-based approaches do not preserve the original structure of microbial communities, they provide valuable information on the fraction of bacteria capable of rapid growth under favourable conditions, which may be particularly relevant in the context of environmental reactivation and exposure scenarios (Shepelin et al. 2017).

The detection of clinically relevant genera such as *Escherichia*, *Salmonella* and *Enterobacter* supports the interpretation that flood-derived sludge can act as a reservoir of bacteria with potential pathogenic relevance. Members of the Enterobacterales (including Enterobacteriaceae and Morganellaceae) are widely recognised as indicators of faecal contamination and sanitation system failure, and their abundance has been shown to increase markedly following flooding events (Kelman et al. 2011; Lynch and Shaman 2023). Although this study does not include pre-flood baseline data, the observed taxonomic composition and high culturable loads strongly suggest substantial microbial input and amplification associated with the flooding episode.

Culture-based methods did not allow the recovery of *Vibrio* spp., despite targeted enrichment strategies. This observation is consistent with the ecological preference of vibrios for marine or brackish water environments rather than freshwater sediments (Garay et al. 1985). However, weak qPCR signals for *V. vulnificus* indicate that this pathogen may be present at very low abundance or may persist in a viable but non-culturable (VBNC) state. This observation is consistent with previous reports describing the persistence of *Vibrio* spp. under environmental stress conditions and highlights the limitations of relying exclusively on culture-based detection (Fernández-Juárez et al. 2024; Sinigalliano et al. 2007). Furthermore, the detection of vibrios in nearby aquatic environments, including coastal and lagoon systems, supports the plausibility of their transient introduction into flood-affected inland areas (Carmona-Salido et al. 2026; Salvador-Clavell et al. 2025; Mas-Coma et al. 2025).

Beyond taxonomic identification, the functional characterization of isolates provides additional insight into their ecological and clinical relevance (Chenhaka et al. 2023; Reyes-Rodríguez et al. 2025). These identified taxa are well-recognised causes of opportunistic and healthcare-associated infections, particularly in immunocompromised individuals (Ferranti et al. 2018; Huang et al. 2021; Nguyen et al. 2022; Ugwu et al. 2025; Zhao and Alexander 2021). The widespread detection of extracellular enzymatic activities and biofilm formation suggests a strong capacity for environmental persistence and colonization of surfaces (Wang et al. 2025; Ugwu et al. 2025). Furthermore, the identification of selected isolates displaying haemolytic activity, resistance to human serum, and multidrug resistance underscores the presence of bacterial phenotypes associated with increased virulence and survival in host environments. In particular, resistance to human serum is functionally relevant because it reflects the ability of bacteria to evade complement-mediated killing, a key trait linked to bloodstream survival and septicemic potential. (Hernández-Cabanyero et al. 2019). That intermediate profile suggests emerging resistance mechanisms or moderate gene expression. The observed gradient, from baseline susceptibility to last-resort antibiotic resistance, highlights the environmental selective pressure and the high risk of horizontal gene transfer. The convergence of these traits in a single strain is particularly concerning, suggesting the potential for invasive behaviour and severe systemic infection, such as sepsis, especially in susceptible or immunocompromised hosts.

Although these phenotypic traits have been previously described in clinical and environmental isolates, their detection in bacteria recovered from post-flood sludge highlights the convergence of environmental contamination and clinically relevant bacterial features within a single matrix. In particular, the identification of an *E. coli* isolate combining multiple virulence-associated traits, resistance to human serum, and antibiotic resistance supports the hypothesis that such environments may contribute to the emergence or dissemination of potentially invasive strains (Wang et al. 2025). Moreover, the presence of *E. coli* in these samples reinforces the evidence of faecal contamination associated with the flooding event and highlights its relevance as an indicator of sanitary risk (Lynch and Shaman 2023).

This study has several limitations that should be acknowledged. First, sampling was performed at a single time point, which precludes direct assessment of temporal dynamics and does not allow definitive conclusions regarding long-term persistence. Second, the use of enrichment steps, while necessary to recover viable bacteria from complex matrices, introduces biases that prevent accurate quantification of microbial abundance in the original samples (Shepelin et al. 2017). Accordingly, Ct values obtained after enrichment should be interpreted as indicators of relative detection rather than absolute quantification. Third, phenotypic characterization was performed in a subset of isolates, which limits the generalisation of these findings to the entire microbial community. Despite these limitations, our results provide evidence that flood-derived sludge can function as a reservoir. From a public health perspective, this finding is particularly relevant, as such environments may facilitate human exposure through direct contact, aerosolisation, or secondary contamination of water systems (Cann et al. 2013; Sinigalliano et al. 2007).

In the context of climate change, where extreme precipitation events are expected to increase in frequency and intensity in Mediterranean regions (AEMET 2025; Erickson et al. 2019), the accumulation of contaminated sludge in inhabited areas represents an emerging and under-recognised risk (Ahmed et al. 2018). Our findings support the need to incorporate microbiological surveillance, including viability-based and functional approaches, into post-flood risk assessment frameworks.

Overall, this study highlights the importance of considering flood-derived sediments not only as passive environmental residues, but as dynamic microbial reservoirs with potential implications for human and animal health under a One Health perspective.

These findings further support the integration of environmental surveillance into risk assessment strategies following extreme climatic events.

## Conclusion

This study demonstrates that flood-derived sludge deposited after the October 2024 DANA event constitutes an active reservoir of viable bacteria with potential public health relevance. The combination of high bacterial loads, the dominance of opportunistic pathogenic genera, and the presence of multiple virulence- and resistance-associated traits—including strong biofilm formation, extracellular enzymatic activity, resistance to human serum, and reduced antimicrobial susceptibility—indicates that these sediments may represent a persistent sanitary risk rather than a transient post-disaster by-product.

Notably, the identification of an *E. coli* isolate combining resistance to human serum, multiple virulence-associated phenotypes, and multidrug resistance highlights the potential presence of bacteria with increased invasive capacity within these environments.

Our findings underscore the need to explicitly incorporate microbiological risk assessment into post-flood management and remediation strategies, particularly in inhabited areas where human exposure is likely. Systematic monitoring of disaster-associated sediments should therefore be considered an essential component of public health surveillance following extreme hydrometeorological events.

Future studies should employ high-resolution approaches, such as whole-genome sequencing and metagenomics, to improve taxonomic resolution and to assess the persistence, evolution, and potential horizontal transfer of virulence and antimicrobial resistance determinants in post-flood environments.

## Supplementary Information

Below is the link to the electronic supplementary material.

Supplementary Material 1 (DOCX 50.5 KB)

## Data Availability

No datasets were generated or analysed during the current study.
