# Supplementary material for: Flood-derived sludge as a reservoir of viable opportunistic pathogens after the October 2024 DANA in Valencia (Spain)
Source: Int Microbiol. 2026 Apr 16;29(6):851–62. doi: 10.1007/s10123-026-00821-4 (PMC13468984; doi:10.1007/s10123-026-00821-4)
Supplement: Supplementary file 1 [file 10123_2026_821_MOESM1_ESM.docx]

**Flood-Derived Sludge as a Reservoir of Viable Opportunistic Pathogens after the October 2024 DANA in Valencia (Spain)**

Marina Pérez-Lara^a^, Pablo Ibáñez-Payá^a^, David Ruiz-Platón^b^, Héctor Carmona-Salido^a^, José Juan Mateo^b^, Carmen Amaro^a^, Rubén Salvador-Clavell^a^

^a^Instituto Universitario de Investigación en Biotecnología y Biomedicina, Universitat de València, Burjassot, Spain

bDepartamento de Microbiología y Ecología, Universitat de València, Burjassot, Spain

Corresponding author e-mail: carmen.amaro@uv.es

**Supplementary Table 1. Primers used for quantitative PCR (qPCR**), including target genes, sequences and amplicon sizes (degenerate bases follow IUPAC nomenclature).

| Microorganism | Gene | Sequence | Product (bp) | Reference |
| --- | --- | --- | --- | --- |
| *Salmonella* sp. | *bipA* | F: 5'-GGTCTGCTGTACTCCACCTTCAG-3' | 86 | Moraes et al. (2016) |
|  |  | R: 5'-TTGGAGATCAGTACGCCGTTCT-3' |  |  |
| *Escherichia coli* | *uidA* | F: 5'-GTCCAAAGCGGCGATTTG-3' | 59 | Kacprzak et al. (2015) |
|  |  | R: 5'-GAGGCCAGAAGTTCTTTTTCCA-3' |  |  |
| *Enterobacter cloacae* | *CIII* | F: 5'-CTGCGTCAGATCGTGTCCAA-3' | 90 | Ohad et al. (2014) |
|  |  | R: 5'-CGTTGTAACCGTAGTTACCTTCACC-3' |  |  |
| *Klebsiella* sp. | *kp4432* | F: 5'-CGGCATCGTATGGGTCAAT-3' | 128 | Patole et al. (2021) |
|  |  | R: 5'-CCGACTTCAGCTCCGTATAATC-3' |  |  |
| *Proteus vulgaris* | *blaB* | F: 5'-TCGTAAAGAGCCTGAATTAA-3' | 229 | Yang et al. (2020) |
|  |  | R: 5'-ATCACCACTACCGGTTTTATC-3' |  |  |
| *Shigella* sp. | *ipaH* | F: 5'-GCTGACCGGCTGAAAGCATC-3' | 131 | This study |
|  |  | R: 5'-AATCGCAGGGCCAGTACCTC-3' |  |  |
| *Vibrio cholerae* | *viuB* | F: 5'-TCGGTATTGTCTAACGGTAT-3' | 77 | Nasreen et al. (2020) |
|  |  | R: 5'-CGATTCGTGAGGGTGATA-3' |  |  |
| *V. cholerae (toxigenic)* | *ctxA* | F: 5'-CAGGTGGTCTTATGCCAAG-3' | 106 |  |
|  |  | R: 5'-CTAACAAATCCCGTCTGAGTT-3' |  |  |
| *V. cholerae* (O1) | *rfbO1* | F: 5'-GTAAAGCAGGATGGAAACATATTC-3' | 113 |  |
|  |  | R: 5'-TGGGCTTACAAACTCAAGTAAG-3' |  |  |
| *V. vulnificus* | *vvhA* | F: 5'-TGTTTATGGTGAGAACGGTGACA-3' | 100 | Garrido-Maestu et al. (2014) |
|  |  | R: 5'-TTCTTTATCTAGGCCCCAAACTTG-3' |  |  |
| *V. parahaemolyticus* | *tlh* | F: 5'-ACTCAACACAAGAAGAGATCGACAA-3' | 208 | Garrido-Maestu et al. (2014) |
|  |  | R: 5'-GATGAGCGGTTGATGTCCAA-3' |  |  |
| *V. metoecus* | *mcp* | F: 5'-GCAGTCTCTTGCCGAAACACTA-3' | 81 | Nasreen et al. (2020) |
|  |  | R: 5'-ATGAACAGCTTATCTTGCCATTC-3' |  |  |
| *Clostridium tetani* | *tenT* | F: 5'-ATGCGCCATCGTATACTAAC-3' | 187 | Guo et al. (2022) |
|  |  | R: 5'-CCATCTTTCGGATAACCTACA-3' |  |  |
| *C. botulinun* type A | *BoNTA* | F: 5'-CGAAATGGTTATGGCTCTACTCAA-3' | 107 | Johnson et al (2014) |
|  |  | R: 5'-TTGCCTGCACCTAAAAGAGGAT-3' |  |  |
| *C. botulinun* type B | *BoNTB* | F: 5'- GATTATAAATGGTATACCTTATCTTGGAGATAGAC-3' | 117 |  |
|  |  | R: 5'-CGCTCCACTTCTCCTGGATTAC -3' |  |  |
| *C. botulinun* type C | *BoNTC* | F: 5'- TGGTGCATTTGTGATTTATAGTAAGGT-3' | 110 |  |
|  |  | R: 5'-CACGTTCCYATCATCCATTCATATG -3' |  |  |
| *C. perfringens* | *gyrB* | F: 5'-GGAAACAGTCTTATAAAAGAGGGGC-3' | 82 | Magdesian et al. (2022) |
|  |  | R: 5'-TGAACTTTAGTTCCACTACCTTCAGC-3' |  |  |

**Supplementary Table 2. Primers used for species-specific conventional PCR identification based on the results obtained by** 16S rRNA Sanger sequencing. Target genes, amplicon sizes and melting temperature (Tm) are also shown.

| Microorganism | Gene | Sequence | Product (bp) | Tm (ºC) | Reference |
| --- | --- | --- | --- | --- | --- |
| *Aeromonas caviae* | *gyrB* | F: 5'-TGCTGCTGACCATCCGC-3' | 79 | 67 | Persson et al. (2015) |
|  |  | R: 5'-GGTGCCTGCGGCTCG-3' |  |  |  |
| *A. hydrophila* | *gyrB* | AGTCTGCCGCCAGTGGC-3 | 144 |  |  |
|  |  | R: 5'-CRCCCATCGCCTGTTCG-3' |  |  |  |
| *A. media* | *gyrB* | F: 5'-GGCCAAGCGTCTGCGT-3' | 99 |  |  |
|  |  | R: 5'-CGCCCTCGTAGCAGAAGTGA-3' |  |  |  |
| *A. veronii* | *rpoB* | F: 5'-CGTGCCGGCTTTGAAGTC-3' | 224 |  |  |
|  |  | R: 5'-GATCACGTACTTGCCTTCTTCAATA-3' |  |  |  |
| *Escherichia coli* | *cdgR* | F: 5'-CCAGGCAAAGAGTTTATGTTGA-3' | 212 | 57 | Lindsey et al. (2017) |
|  |  | R: 5'-GCTATTTCCTGCCGATAAGAGA-3' |  |  |  |
| *E. albertii* | *EAKF1_ch4033* | F: 5'-GTAAATAATGCTGGTCAGACGTTA-3' | 393 |  |  |
|  |  | R: 5'-AGTGTAGAGTATATTGGCAACTTC-3' |  |  |  |
| *E. fergusonii* | EFER_0790 | F: 5'-AGATTCACGTAAGCTGTTACCTT-3' | 575 |  |  |
|  |  | R: 5'-CGTCTGATGAAAGATTTGGGAAG-3' |  |  |  |
| *E. coli* (enteroadherent) | *astA* | F: 5'-TATATCCGAAGGCCCGCATCCAG-3' | 100 |  | Ohmura-Hoshino et al. (2022) |
|  |  | R: 5'-CAGGTCGCGAGTGACGGCTTTG-3' |  |  |  |
|  | *aggT* | F: 5'-GCCTAAAGGATGCCCTGATG-3' | 407 |  |  |
|  |  | R: 5'-TGCTGCTTTGCTCATTCTTG-3' |  |  |  |
| *E. coli* (enterohaemorrhagic) | *stx1* | F: 5'-AGAGGGATAGATCCAGAGGAAGGG-3' | 693 |  |  |
|  |  | R: 5'-AATTGCCCCCAGAGTGGATG-3' |  |  |  |
|  | *stx2* | F: 5'-GTATACGGACAGAGATATCGACCCC-3' | 794 |  |  |
|  |  | R: 5'-CGCTGCAGCTGTATTACTTTCCC-3' |  |  |  |
| *E. coli* (enteroinvasive) | *ipaH* | F: 5'-CTTTCCGATACCGTCTCTGC-3' | 596 |  |  |
|  |  | R: 5'-CACCCTCTGAGAGTACTCATTCTCC-3' |  |  |  |
|  | *invE* | F: 5'-GAAATTCTGGATGGCACTCGTAGAAG-3' | 235 |  |  |
|  |  | R: 5'-CTTTCGCGCGAGACAGATTCTCTT-3' |  |  |  |
| *E. coli* (enteropaghotenic) | *bfpA* | F: 5'-CTACCAGTCTGCGTCTGATTCC-3' | 326 |  |  |
|  |  | R: 5'-CGTAGCCTTTCGCTGAAGTACC-3' |  |  |  |
|  | *eaeA* | F: 5'-CTTCAGTCGCGATCTCTGAACG-3' | 1001 |  |  |
|  |  | R: 5'-GGTAGTCTTGTGCGCTTTGGCT-3' |  |  |  |
| *E. coli* (enterotoxigenic) | *elt* | F: 5'-TATACCGTGCTGACTCTAGACCCC-3' | 494 |  |  |
|  |  | R: 5'-CGGTGGGAAACCTGCTAATC-3' |  |  |  |
|  | *sth* | F: 5'-CTTTCTGTATTGTCTTTTTCACCTTTC-3' | 172 |  |  |
|  |  | R: 5'-GCAGGATTACAACACAATTCACAGC-3' |  |  |  |
| *Enterobacter cloacae* | ECL_01098 | F: 5'-TGAAAACCTTATCCGCGA-3' | 397 | 57 | Ji et al. (2021) |
|  |  | R: 5'-GGCAGGCTGGAAGATAAA-3' |  |  |  |
| *E. hormaechei* | *pqqB* (LI64_09975) | F: 5'-AACTGTCAGGGTTTGCGC-3' | 487 |  |  |
|  |  | R: 5'-CAAACAGCGCCACGTTAT-3' |  |  |  |
| *E. roggenkampii* | EGY04_15785 | F: 5'-ATCAGCATCGGGATCGGT-3' | 1132 |  |  |
|  |  | R: 5'-GCTTTTGAACAAACTCAGCATA-3' |  |  |  |
| *E. kobei* | *gspE* (BFV64_21570) | F: 5'-GGCATTGCCTTACAAGGAG-3' | 1403 |  |  |
|  |  | R: 5'-GTCACCCGCAGAATTTCT-3' |  |  |  |
| *Shigella* spp*.* | NC_004337: 559294-559452 | F: 5'-TCCGTCATGCTGGATGAACGATGT-3' | 159 | 60 | Ranjbar et al. (2014) |
|  |  | R: 5'-ACAGTTCAGGATTGCCCGAGACACA-3' |  |  |  |
| *S. boydii* | NC_007613: 130607-1360854 | F: 5'-TCTGATGTCACTCTTTGCGAGT-3' | 248 |  |  |
|  |  | R: 5'-GAATCCGGTACCCGTAAGGT-3' |  |  |  |
| *S. sonnei* | NC_007384: 1665725-1666227 | F: 5'-AATGCCGTAAGGAATGCAAG-3' | 503 |  |  |
|  |  | R: 5'-CTTGAAGGAGATTCGCTGCT-3' |  |  |  |
| *S. flexneri* | NC_004337: 1412593-1412906 | F: 5'-ACCGGTTATGAACCCTCCAT-3' | 314 |  |  |
|  |  | R: 5'-TGGTGCTTGTTGAGCAACTC-3' |  |  |  |

**Supplementary Table 3. Antibiotic disks and concentrations used in antimicrobial susceptibility testing.**

| Antibiotic | Concentration (µg) | Manufacturer |
| --- | --- | --- |
| Amoxicillin/Clavulanic acid | 20/10 | BD BBL Sensi-Disc, BD (USA) |
| Aztreonam | 30 | Oxoid (UK) |
| Cefoxitin | 30 | Oxoid (UK) |
| Ceftazidime | 30 | Liofilchem (Italy) |
| Ciprofloxacin | 5 | BD BBL Sensi-Disc, BD (USA) |
| Ertapenem | 10 | Oxoid (UK) |
| Netilmicin | 30 | Oxoid (UK) |
| Piperacillin | 100 | Oxoid (UK) |
| Sulfamethoxazole/Trimethoprim | 25 | Oxoid (UK) |

**Supplementary Table 4. BLAST-based identification of bacterial isolates based on partial 16S rRNA gene sequencing.** Results of partial 16S rRNA gene sequencing are displayed, detailing the identification achieved through BLAST analysis and the corresponding percentage of sequence identity (hit).

| **Isolate** | **Genus** | **Specie(s)** | **Subject accession** | **% identify** | **E-value** |
| --- | --- | --- | --- | --- | --- |
| 1 | *Citrobacter* | *pasteurii* | NR_178769.1 | >99.07 | 0.00 |
|  |  | *braakii* | NR_117750.1 |  |  |
|  |  | *freundii* | NR_117752.1 |  |  |
| 2 | *Pseudomonas* | *kurunegalensis* | NR_178288.1 | >99.90 | 0.00 |
|  |  | *laurentiana* | NR_179898.1 |  |  |
|  |  | *vlassakiae* | NR_178289.1 |  |  |
| 3 | *Pseudomonas* | *promysalinigenes* | NR_178291.1 | >99.40 | 0.00 |
|  |  | *kurunegalensis* | NR_178288.1 |  |  |
|  |  | *fortuita* | NR_197682.1 |  |  |
| 4 | *Aeromonas* | *media* | NR_036911.2 | >99.70 | 0.00 |
|  |  | *hydrophila* | NR_118944.1 |  |  |
| 5 | *Aeromonas* | *intestinalis* | NR_179576.1 | >98.31 | 0.00 |
|  |  | *caviae* | NR_104824.1 |  |  |
|  |  | *veronii* | NR_118947.1 |  |  |
| 6 | *Aeromonas* | *rivipollensis* | NR_144574.1 | >99.30 | 0.00 |
|  |  | *taiwanensis* | NR_116585.1 |  |  |
|  |  | *hydriphila* | NR_042518.1 |  |  |
|  |  | *dhakensis* | NR_042155.1 |  |  |
| 7 | *Shigella* | *flexneri* | NR_026331.1 | >99.70 | 0.00 |
|  | *Shigella* | *sonnei* | NR_104826.1 |  |  |
|  | *Escherichia* | *fergusonii* | NR_027549.1 |  |  |
|  | *Escherichia* | *coli* | NR_074902.1 |  |  |
| 8 | *Shigella* | *sonnei* | NR_104826.1 | >99.40 | 0.00 |
|  | *Shigella* | *boydii* | NR_104901.1S |  |  |
|  | *Shigella* | *flexneri* | NR_026331.1 |  |  |
|  | *Escherichia* | *fergusonii* | NR_027549.1 |  |  |
| 9 | *Citrobacter* | *freundii* | NR_113340.1 | 99.70 | 0.00 |
| 10 | *Aeromonas* | *media* | NR_036911.2 | >99.70 | 0.00 |
|  |  | *hydrophila* | NR_074841.1 |  |  |
| 11 | *Aeromonas* | *media* | NR_036911.2 | >99.80 | 0.00 |
|  |  | *hydrophila* | NR_118944.1 |  |  |
| 12 | *Pseudomonas* | *laurentiana* | NR_179898.1 | >99.80 | 0.00 |
|  |  | *vlassakiae* | NR_178289.1 |  |  |
|  |  | *promysalinigenes* | NR_178291.1 |  |  |
|  |  | *monteilii* | NR_114224.1 |  |  |
| 13 | *Pseudomonas* | *glycinis* | NR_181729.1 | >99.48 | 0.00 |
|  |  | *koreensis* | NR_025228.1 |  |  |
|  |  | *reinekei* | NR_042541.1 |  |  |
|  |  | *moraviensis* | NR_043314.1 |  |  |
| 14 | *Stutzerimonas* | *chloritidismutans* | NR_115115.1 | >99.29 | 0.00 |
|  | *Pseudomonas* | *knackmussii* | NR_117756.1 |  |  |
|  | *Stutzerimonas* | *kunmingensis* | NR_133828.1 |  |  |
|  | *Stutzerimonas* | *xanthomarina* | NR_041044.1 |  |  |
| 15 | *Citrobacter* | *pasteurii* | NR_178769.1 | >99.01 | 0.00 |
|  |  | *gillenii* | NR_041697.1 |  |  |
|  |  | *braakii* | NR_117750.1 |  |  |
|  |  | *freundii* | NR_113340.1 |  |  |
| 16 | *Aeromonas* | *enteropelogenes* | NR_116026.1 | >99.70 | 0.00 |
|  |  | *hydrophila* | NR_119190.1 |  |  |
|  |  | *taiwanensis* | NR_116585.1 |  |  |
|  |  | *caviae* | NR_029252.1 |  |  |
| 17 | *Acinetobacter* | *junii* | NR_117623.1 | >98.50 | 0.00 |
|  |  | *modestus* | NR_148845.1 |  |  |
|  |  | *plantarum* | NR_178762.1 |  |  |
| 18 | *Enterobacter* | *bugadensis* | NR_148649.1 | >99.64 | 0.00 |
|  |  | *asburiae* | NR_024640.1 |  |  |
|  |  | *cloacae* | NR_118568.1 |  |  |
| 19 | *Acinetobacter* | *junii* | NR_117623.1 | >98.52 | 0.00 |
|  |  | *modestus* | NR_148845.1 |  |  |
|  |  | *plantarum* | NR_178762.1 |  |  |
| 20 | *Escherichia* | *fergusonii* | NR_074902.1 | >98.90 | 0.00 |
|  | *Shigella* | *boydii* | NR_104901.1 |  |  |
|  | *Shigella* | *flexneri* | NR_026331.1 |  |  |
|  | *Escherichia* | *coli* | NR_114042.1 |  |  |
| 21 | *Escherichia* | *fergusonii* | NR_074902.1 | >99.40 | 0.00 |
|  | *Shigella* | *flexneri* | NR_026331.1 |  |  |
|  | *Escherichia* | *coli* | NR_114042.1 |  |  |
|  | *Escherichia* | *fergusonii* | NR_027549.1 |  |  |
|  | *Shigella* | *sonnei* | NR_104826.1 |  |  |
| 22 | *Citrobacter* | *freundii* | NR_113340.1 | >99.50 | 0.00 |
|  |  | *braakii* | NR_028687.1 |  |  |
|  |  | *murliniae* | NR_028688.1 |  |  |
| 23 | *Enterobacter* | *quasihormaechei* | NR_180451.1 | >99.90 | 0.00 |
|  |  | *hormaechei* | NR_126208.1 |  |  |
|  |  | *cloacae* | NR_118568.1 |  |  |
| 24 | *Citrobacter* | *braakii* | NR_028687.1 | >99.18 | 0.00 |
|  |  | *freundii* | NR_117752.1 |  |  |
| 25 | *Citrobacter* | *freundii* | NR_113340.1 | >99.50 | 0.00 |
|  |  | *braakii* | NR_113596.1 |  |  |
|  |  | *murliniae* | NR_028688.1 |  |  |
| 26 | *Shigella* | *flexneri* | NR_026331.1 | >99.30 | 0.00 |
|  | *Shigella* | *sonnei* | NR_104826.1 |  |  |
|  | *Escherichia* | *fergusonii* | NR_027549.1 |  |  |
|  | *Escherichia* | *fergusonii* | NR_074902.1 |  |  |
| 27 | *Escherichia* | *fergusonii* | NR_074902.1 | >98.82 | 0.00 |
|  | *Shigella* | *flexnerii* | NR_026331.1 |  |  |
|  | *Escherichia* | *coli* | NR_114042.1 |  |  |
| 28 | *Aeromonas* | *allosaccharophila* | NR_025945.2 | >99.20 | 0.00 |
|  |  | *veronii* | NR_118947.1 |  |  |
|  |  | *rivuli* | NR_119045.1 |  |  |
| 29 | *Pseudomonas* | *promysalinigenes* | NR_178291.1 | 99.40 | 0.00 |
|  |  | *kurunegalensis* | NR_178288.1 |  |  |
|  |  | *fortuita* | NR_197682.1 |  |  |
| 30 | *Stutzerimonas* | *chloritidismutans* | NR_115115.1 | >99.19 | 0.00 |
|  | *Pseudomonas* | *knackmussii* | NR_117756.1 |  |  |
|  | *Stutzerimonas* | *kunmingensis* | NR_133828.1 |  |  |
|  | *Stutzerimonas* | *xanthomarina* | NR_041044.1 |  |  |

**References**

Garrido-Maestu A, Chapela M.-J, Peñaranda E, Vieites JM, Cabado AG (2014) In-house validation of novel multiplex real-time PCR gene combination for the simultaneous detection of the main human pathogenic vibrios (*Vibrio cholerae*, *Vibrio parhaemolyticus*, and *Vibrio vulnificus*). Food Control 37:371–379. <https://doi.org/10.1016/j.foodcont.2013.09.026>

Guo M, Feng P, Zhang L, Feng C, Fu J, Pu X, Liu F (2022) Rapid Detection of *Clostridium tetani* by Recombinase Polymerase Amplification Using an Exo Probe. J Microbiol Biotechnol 32(1):91-98. https://doi.org/10.4014/jmb.2109.09022

Ji Y, Wang P, Xu T, Zhou Y, Chen R, Zhu H, Zhou K (2021) Development of a One-Step Multiplex PCR Assay for Differential Detection of Four species (*Enterobacter cloacae*, *Enterobacter hormaechei*, *Enterobacter roggenkampii*, and *Enterobacter kobei*) Belonging to *Enterobacter cloacae* Complex With Clinical Significance. Frontiers in cellular and infection microbiology 11:677089. https://doi.org/10.3389/fcimb.2021.677089

Johnson AL, McAdams-Gallagher SC, Sweeney RW (2014) Quantitative real-time PCR for detection of neurotoxin genes of *Clostridium botulinum* types A, B and C in equine samples. Veterinary journal 199(1):157–161. https://doi.org/10.1016/j.tvjl.2013.10.023

Kacprzak M, Fijałkowski K, Grobelak A, Rosikoń K, Rorat A (2015) *Escherichia coli* and *Salmonella* spp. Early Diagnosis and Seasonal Monitoring in the Sewage Treatment Process by EMA-qPCR Method. Pol J Microbiol 64(2):143–148. https://doi.org/10.33073/pjm-2015-021

Lindsey RL, Garcia-Toledo L, Fasulo D, Gladney LM, Strockbine N (2017) Multiplex polymerase chain reaction for identification of *Escherichia coli*, *Escherichia albertii* and *Escherichia fergusonii*. J Microbiol Methods 140:1–4. <https://doi.org/10.1016/j.mimet.2017.06.005>

Magdesian KG, Barnum S, Pusterla N (2022) Fecal PCR testing for detection of *Clostridium perfringens* and *Clostridioides* difficile toxin genes and other pathogens in foals with diarrhea: 28 cases. Journal of veterinary diagnostic investigation: official publication of the American Association of Veterinary Laboratory Diagnosticians, Inc 34(3):396–401. https://doi.org/10.1177/10406387211047529

Moraes DMC, Duarte SC, Bastos TSA, Rezende CLG, Leandro NSM, Café MB, Stringhini JH, Andrade MA (2016). Detection of *Salmonella* spp. by conventional bacteriology and by quantitative polymerase-chain reaction in commercial egg structures. Braz J Poult Sci 18(1):117–124. <https://doi.org/10.1590/18069061-2015-0063>

Nasreen T, Hussain NAS, Islam MT, Orata FD, Kirchberger PC, Case RJ, Alam M, Yanow SK, Boucher YF (2020) Simultaneous Quantification of *Vibrio metoecus* and *Vibrio cholerae* with Its O1 Serogroup and Toxigenic Subpopulations in Environmental Reservoirs. Pathogens 9(12):1053. <https://doi.org/10.3390/pathogens9121053>

Ohad S, Block C, Kravitz V, Farber A, Pilo S, Breuer R, Rorman E (2014) Rapid identification of *Enterobacter hormaechei* and *Enterobacter cloacae* genetic cluster III. J Appl Microbiol 116(5):1315–1321. <https://doi.org/10.1111/jam.12439>

Ohmura-Hoshino M, Miyaki Y, Yashima S (2022) A one-step multiplex PCR-based assay for simultaneous detection and classification of virulence factors to identify five diarrheagenic *E. coli* pathotypes. Heliyon *8*(8):e10231. https://doi.org/10.1016/j.heliyon.2022.e10231

Patole S, Rout M, Mohapatra H (2021) Identification and validation of reference genes for reliable analysis of differential gene expression during antibiotic induced persister formation in *Klebsiella pneumoniae* using qPCR. J Microbiol Methods 182:106165. <https://doi.org/10.1016/j.mimet.2021.106165>

Persson S, Al-Shuweli S, Yapici S, Jensen JN, Olsen KE (2015) Identification of clinical aeromonas species by *rpoB* and *gyrB* sequencing and development of a multiplex PCR method for detection of *Aeromonas hydrophila*, *A. caviae*, *A. veronii*, and *A. media*. J Clin Microbiol 53(2):653–656. <https://doi.org/10.1128/JCM.01963-14>

Ranjbar R, Afshar D, Mehrabi Tavana A, Najafi A, Pourali F, Safiri Z, Sorouri Zanjani R, Jonaidi Jafari N (2014) Development of Multiplex PCR for Simultaneous Detection of Three Pathogenic *Shigella* Species. Iranian journal of public health 43(12):1657–1663

Yang R, Xu G, Wang X, Qing Z, Fu L (2020) Establishment and Application of a Dual TaqMan Real-Time PCR Method for *Proteus Mirabilis* and *Proteus Vulgaris*. Pol J Microbiol 69(3):293–300. <https://doi.org/10.33073/pjm-2020-032>
